# Widespread presence of the pathogenic fungus *Batrachochytrium dendrobatidis* in wild amphibian communities in Madagascar

**DOI:** 10.1038/srep08633

**Published:** 2015-02-26

**Authors:** Molly C. Bletz, Gonçalo M. Rosa, Franco Andreone, Elodie A. Courtois, Dirk S. Schmeller, Nirhy H. C. Rabibisoa, Falitiana C. E. Rabemananjara, Liliane Raharivololoniaina, Miguel Vences, Ché Weldon, Devin Edmonds, Christopher J. Raxworthy, Reid N. Harris, Matthew C. Fisher, Angelica Crottini

**Affiliations:** 1 Department of Biology, James Madison University, Harrisonburg, VA 22807, USA; 2 Technische Universität Braunschweig, Division of Evolutionary Biology, Zoological Institute, Mendelssohnstr. 4, 38106 Braunschweig, Germany; 3 Durrell Institute of Conservation and Ecology, School of Anthropology and Conservation, University of Kent, Canterbury, Kent CT2 7NR, UK; 4 Institute of Zoology, Zoological Society of London, Regent's Park, London NW1 4RY, UK; 5 Centre for Ecology, Evolution and Environmental Changes (CE3C), Faculdade de Ciências da Universidade de Lisboa, Bloco 2, Piso 5, Campo Grande, 1749-016 Lisbon, Portugal; 6 Museo Regionale di Scienze Naturali, Via G. Giolitti, 36, I-10123, Torino, Italy; 7 IUCN SSC Amphibian Specialist Group-Madagascar, 101 Antananarivo, Madagascar; 8 CNRS-Guyane, USR 3456, 2 avenue Gustave Charlery, 97300 Cayenne, Guyane Française; 9 Station d'écologie expérimentale du CNRS à Moulis, USR 2936, 2 route du CNRS, 09200 Moulis, France; 10 UFZ – Helmholtz Centre for Environmental Research, Department of Conservation Biology, Permoserstr. 15, 04318 Leipzig, Germany; 11 EcoLab (Laboratoire Ecologie Fonctionnelle et Environnement), CNRS/Université de Toulouse; UPS, INPT; 118 route de Narbonne, 31062 Toulouse, France; 12 Département de Biologie Animale et Ecologie, Faculté des Sciences, University of Mahajanga, Ambondrona, B.P. 652, Mahajanga 401, Madagascar; 13 University of Antananarivo, BP 906, Antananarivo 101, Antananarivo, Madagascar; 14 Unit for Environmental Sciences and Management, North-West University, Private Bag X6001, Potchefstroom 2520, South Africa; 15 Association Mitsinjo, Lot 104 A Andasibe Gare, Andasibe 514, Madagascar; 16 Department of Herpetology, American Museum of Natural History, Central Park West at 79^th^ St. New York, NY 10024, USA; 17 Department of Infectious Disease Epidemiology, Imperial College London, W2 1PG, UK; 18 CIBIO Research Centre in Biodiversity and Genetic Resources, InBIO, Universidade do Porto, Campus Agrário de Vairão, Rua Padre Armando Quintas, N° 7, 4485-661 Vairão, Vila do Conde, Portugal

## Abstract

Amphibian chytridiomycosis, an emerging infectious disease caused by the fungus *Batrachochytrium dendrobatidis* (*Bd*), has been a significant driver of amphibian declines. While globally widespread, *Bd* had not yet been reported from within Madagascar. We document surveys conducted across the country between 2005 and 2014, showing *Bd*'s first record in 2010. Subsequently, *Bd* was detected in multiple areas, with prevalence reaching up to 100%. Detection of *Bd* appears to be associated with mid to high elevation sites and to have a seasonal pattern, with greater detectability during the dry season. Lineage-based PCR was performed on a subset of samples. While some did not amplify with any lineage probe, when a positive signal was observed, samples were most similar to the Global Panzootic Lineage (*Bd*GPL). These results may suggest that *Bd* arrived recently, but do not exclude the existence of a previously undetected endemic *Bd* genotype. Representatives of all native anuran families have tested *Bd*-positive, and exposure trials confirm infection by *Bd* is possible. *Bd*'s presence could pose significant threats to Madagascar's unique “megadiverse” amphibians.

Amphibian population declines and extinctions are occurring at unprecedented rates^1^. Multiple anthropogenic factors including habitat destruction and alteration, introduction of alien species and over-exploitation are linked to the global declines of amphibians. Chytridiomycosis, an emerging infectious disease caused by the pathogen *Batrachochytrium dendrobatidis* (*Bd*), is also recognized as playing a significant role in the rapid declines and extinctions of amphibians around the world^2^,^3^. *Bd* has been detected in over 500 species worldwide (http://www.bd-maps.net/), and at least 200 species have declined as a result of chytrid infection^4^. *Bd* has decimated amphibian populations in the Neotropics, Australian Wet Tropics, the western USA, Europe, and east Africa^5^,^6^,^7^,^8^. For example, in the Neotropics, approximately 67% (110 species) of the genus *Atelopus* disappeared across their range^9^, and when *Bd* arrived in Panama, 41% of the amphibian diversity was lost from the highland site of El Copé^10^. The pathogen's ability to infect numerous host species and spread rapidly through amphibian assemblages and on a global scale make it the greatest disease threat to biodiversity at the current time^11^. Despite the occurrence of *Bd* on almost every continent, there are regions of the world that are considered pathogen-free, including Papua New Guinea^12^ and Madagascar. In Madagascar, comprehensive surveys for *Bd* were conducted in 2005–2010 with no detection of the pathogen^13^,^14^,^15^,^16^, despite much of the eastern rainforest being climatically suitable for *Bd*^17^,^37^.

Madagascar harbours an extraordinary amphibian diversity, with more than 290 described species and well over 200 undescribed candidate species of frogs belonging to five independent radiations^18^,^19^. Except for two introduced species, all Malagasy amphibians are endemic to the island which therefore hosts a considerable proportion of the currently ca. 7300 amphibian species inhabiting the world^20^. Many Malagasy rainforest sites hold numerous sympatric amphibian species^21^ and an extraordinary density of adults and tadpoles^22^. Comparatively high frog diversity also occurs in apparently hostile dry habitats with temporary pools^23^. The most important threat to Madagascar's amphibians is deforestation, yet some species are also threatened by excessive pet trade collection and the likely effects of climate change and habitat alteration^24^,^25^,^26^,^27^. The introduction of a non-native, virulent lineage of *Bd* would add to the threats posed against Madagascar's unique amphibian communities.

In the past years, numerous activities to meet the challenges of amphibian conservation in Madagascar have occurred. In 2006 the first “A Conservation Strategy for the Amphibians of Madagascar” workshop was organized to develop the “Sahonagasy Action Plan”, a national action plan^28^, resulting later in several actions, including information management and citizen-science initiatives, reserve planning^29^, and in-situ breeding facilities^30^. In 2010, the Chytridiomycosis Working Group (CWG) was established to facilitate chytrid-related research in Madagascar and the Chytrid Emergency Cell (CEC) was created to develop specific protocols to prevent the arrival of *Bd* and to rapidly respond to chytridiomycosis outbreaks in Madagascar. In addition, the National Monitoring Plan (NMP) which biannually surveys for *Bd* across the island at eight selected sites was launched^31^. All these activities started under the premise that *Bd* was absent in Madagascar, according to the surveys published before 2014^13^,^14^,^15^,^16^,^32^; however, recently *Bd* was reported on Malagasy frogs imported to the USA for the pet trade in 2012^33^.

Here we present the first evidence for the widespread presence of *Bd* in wild amphibian populations from *Bd* surveys carried out from 2005–2014 at various sites across the country, and provide preliminary information about the identity of the *Bd* lineage.

## Results

Results are based on the analysis of 4,155 amphibians tested for the presence of *Bd*, 1,113 of which have been presented in previous publications^13^,^14^,^15^,^16^,^32^,^34^. Fifty-two sites across Madagascar were sampled, with the earliest sampling undertaken in 2005. Ninety-nine different sampling events were completed, with a mean sample size of 42 ± 3 SE frogs across these sampling events. For this study we combined all data available to us from 2005–2014. These data are from (i) samples of the National Monitoring Plan, (ii) samples obtained in the context of a skin microbiota study of Madagascar's amphibians, and (iii) samples collected opportunistically. While the data thus do not agree with an ideal sampling design, they do cover all major biomes and elevation zones of the island, as well as both the dry and wet season, allowing for the first assessment of spatial and temporal patterns of *Bd* occurrence and prevalence in Madagascar (see Supplementary Table 1 for more details).

### *Bd* sampling

In 2005–2008, 892 amphibians were sampled in Ambohitantely, An'Ala, Andasibe, Andringitra, Ankarafantsika, Ankaratra, Antananarivo, Isalo, Manakara, Manombo, Masoala, Montagne d'Ambre, and Ranomafana, and all samples tested negative for *Bd*^13^,^14^,^15^.

**Figure 1:**
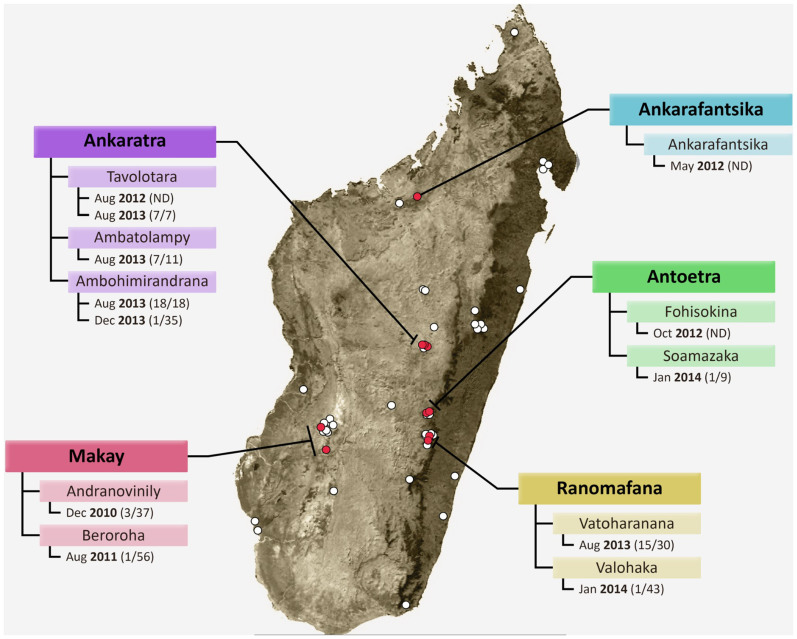
Map of all sites sampled for *Bd.* Circles represent sites of surveys conducted between 2005–2014, and red colouring highlights *Bd*-positive sites. Location name, site name, the month-year of detection and prevalence are provided for each location with *Bd*-positive occurrences. The names of all remaining sites can be found in Supplementary Table 1. ND indicates that prevalence could not be determined due to pooling of collected samples for detection analysis. The base map was obtained from www.worldofmaps.net. Points on the map were generated using QGis 2.0 (Quantum GIS Development Team, 2013) and afterwards edited on Adobe PhotoShop CS6 (Adobe, 2012).

The first record of *Bd* was documented in December 2010 in the Makay Massif (Figure 1 and Table 1). *Bd* was detected in a site locally known as Andranovinily, one of five sampled sites in the Makay Massif. Three individuals out of 37 frogs tested positive, with *Bd* intensities ranging from 0.157–0.273 genome equivalents (GE) (Supplementary Table 1, Figure 1). All positives were from samples of *Mantidactylus* sp. Ca14^19^ (Table 2). In the same year, surveys conducted in Toamasina and Ankaratra did not detect *Bd*^16^,^32^.

In 2011, *Bd* was again detected in Makay. It was found in one sample of *Ptychadena mascareniensis* collected in Beroroha (Figure 1, Table 2), and had a *Bd* intensity of 0.239 GE (Supplementary Table 1). No *Bd* was detected in the 83 samples collected from the site Andranovinily (positive in 2010), nor was it detected at the three other Makay sites visited (Figure 1). Similarly, surveys in other areas, including Ankarafantsika, Toamasina, Antoetra, Andasibe and Mandena, did not yield any *Bd*-positive samples (Figure 1).

In 2012, *Bd* was detected across multiple locations: samples from Ankarafantsika (March), Ankaratra (August), and Antoetra (October) tested positive (Figure 1 and Table 1). The samples were pooled together by site for qPCR analysis to reduce cost and decrease the time needed for sample analysis, and therefore it was not possible to determine prevalence of *Bd* at these locations. At Ankaratra and Antoetra, the positive samples were from *Mantidactylus pauliani* and *M*. sp. Ca48 respectively (Table 2). No information on the species are available from the samples collected in Ankarafantsika. The *Bd* intensities of the pooled samples were 11.0, 21.0, and 2.0 GE for Ankaratra, Antoetra, and Ankarafantsika respectively (Supplementary Table 1). All samples from Andasibe, Ankaratra (May), Antoetra (March), Masoala, Menabe, and Toamasina tested negative for *Bd* (Table 1).

**Figure 2:**
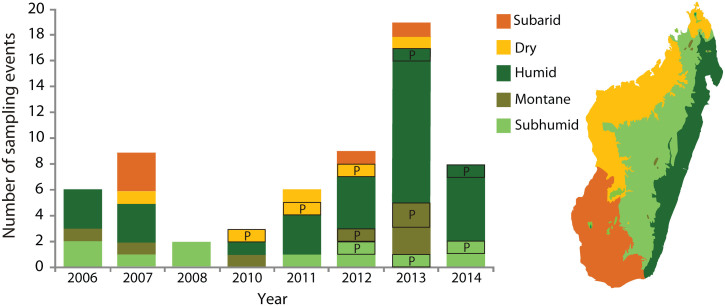
Sampling effort between 2006–2014 for each bioclimatic region of Madagascar. A “P” indicates *Bd*-positive occurrences and the size of the black-outlined boxes represents the number of *Bd*-positive sample events. A map with corresponding colours is included to present the locations of the bioclimatic regions in Madagascar. Only sampling events with PCR-based *Bd* screening are included. The inset map was prepared in Corel Draw software by redrawing an original map from George Schatz.

In 2013, there was increasing sampling effort with 10 different locations surveyed (Figure 2), of which four were surveyed multiple times throughout the year. Samples from Ankaratra and Ranomafana tested positive for *Bd* (Figure 1 and Table 1). More specifically, the Ankaratra region was sampled four times during 2013. In February and June, all Ankaratra samples tested negative for *Bd*, however, subsequently in August, numerous samples tested positive for *Bd*. The prevalence at the Ambohimirandrana and Tavolotara sites within Ankaratra were 100% and the *Bd* intensities ranged from 37.3–167.8 GE and 47.3–95.2 GE respectively (Figure 1, Supplementary Table 1). Five different species tested positive for *Bd*, including *Boophis ankaratra, B*. *goudoti, B. williamsi, Mantidactylus pauliani*, and *M*. sp. Ca19^19^ (Table 2). At Ambatolampy, a ricefield site at the base of the Ankaratra Massif, the prevalence of *Bd* was 63%, and the *Bd* intensity ranged from 15.9–97.6 GE (Figure 1, Supplementary Table 1). The *Bd*-positive samples were collected from *Heterixalus betsileo* and *Ptychadena mascareniensis* (Table 2). In December, one sample collected from *Mantidactylus* sp. Ca19 at Ambohimirandrana tested positive for *Bd*. The *Bd* intensity of this sample was 0.75 GE (Supplementary Table 1). All other Ankaratra sites tested negative for *Bd* in December. At Ranomafana in August, frogs sampled at Vatoharanana tested positive for *Bd*. The prevalence of *Bd* was 50% and the *Bd* intensities ranged from 16.6–145.8 GE (Figure 1, Supplementary Table 1). All samples collected at Andasibe, Ankarafantsika, Makay, Mandena, Masoala (multiple sites), Menabe, Toamasina, and Torotorofotsy tested negative for *Bd* (Table 1).

In early 2014, samples collected in Antoetra and Ranomafana tested positive for *Bd*. At Ranomafana six sites were sampled. One out of 45 samples collected at Valohoaka tested positive for *Bd* (*Boophis reticulatus*; 2.53 GE; Table 2, Supplementary Table 1). In Vatoharanana, which was positive in August 2013, none of 87 sampled individuals were found positive for *Bd*. All samples collected from the other sites in Ranomafana tested negative for *Bd*. In Antoetra, three sites were sampled. *Bd* was detected at one site, Soamazaka, where one out of nine samples tested positive for *Bd* (*Mantella cowani*; 2.88 GE; Table 2, Supplementary Table 1). Samples from Ambohitantely, An'Ala, Andasibe, Fierenana, and Torotorofotsy all tested negative for *Bd*.

**Figure 3:**
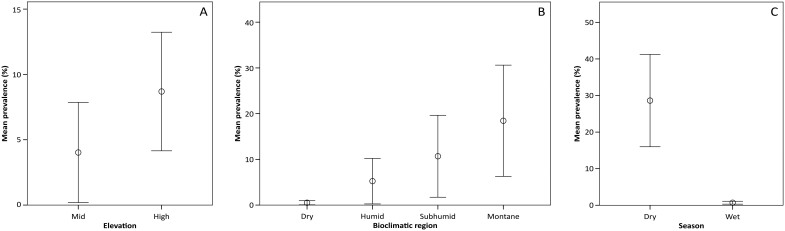
Mean prevalence of *Bd* across positive sites in Madagascar: (A). Prevalence associated with elevation; (B). Prevalence associated with bioclimatic region; (C). Prevalence associated with season. Error bars: +/− 1 SE.

*Bd* prevalence tended to be greater in mid to high elevation sites (800+ meters above sea level^35^) (Figure 3A) and in the montane and subhumid bioclimatic regions (Figure 3B). In addition, there was a trend toward season having an effect on *Bd*'s prevalence across the different locations, with higher values in the dry season (Figure 3C).

### *Bd* lineage in Madagascar

Lineage-specific qPCR was undertaken to determine if the *Bd* present in Madagascar was more closely related to the *Bd*GPL, *Bd*CAPE or *Bd*CH lineages. A subset of samples, that were *Bd* positive with the general ITS probe, were tested with lineage specific probes (Table 3). While not all samples showed positive amplification, the ones that amplified with the lineage specific probes all showed the presence of a *Bd*GPL-like lineage (Table 3). This *Bd*GPL-like lineage may be the true *Bd*GPL, an endemic *Bd*GPL-like, or a non-endemic *Bd*GPL-like lineage. None of the tested samples showed positive amplification with the CAPE-specific or CH-specific probe (Table 3).

### Non-amphibian *Bd* vectors: Crayfish

Crayfish in Antananarivo, Mandraka, and Ranomafana were sampled for *Bd* in January-February 2014. No individuals of either the invasive *Procambarus* sp. or the native *Astacoides* spp. yielded positives for *Bd*.

### Amphibian chytrid fungus infectivity

No data are so far available on the susceptibility of Malagasy frogs to chytridiomycosis. We report preliminary data on infectivity for a series of species of the families Ptychadenidae, Hyperoliidae, and Mantellidae (details in Supplementary Methods). The exposure trials with an isolate of *Bd*GPL confirmed that this *Bd* lineage has the potential to infect individuals of the species *Boophis madagascariensis*, *B. viridis, Heterixalus betsileo, Mantidactylus betsileanus*, and *Ptychadena mascareniensis* (see Supplementary data).

## Discussion

In this study, we document the presence of the amphibian chytrid fungus, *Batrachochytrium dendrobatidis*, in wild populations of amphibians in Madagascar. Previously published surveys (2005–2010) across the country^13^,^14^,^15^,^16^,^32^, in addition to data presented here, show the lack of *Bd* detection prior to 2010. Between 2010 and 2014, *Bd* has been recorded in five different areas of the country: Ankarafantsika (March 2012), Ankaratra (August 2012, August and December 2013), Antoetra, (October 2012, January 2014), Makay (December 2010, August 2011), and Ranomafana (August 2013, January 2014). So far, *Bd* positive samples in Madagascar are distributed over all four families of native Malagasy frogs: Hyperoliidae (*Heterixalus*), Microhylidae (*Platypelis* and *Scaphiophryne*^33^), Ptychadenidae (*Ptychadena*) and Mantellidae (*Boophis, Gephyromantis, Mantella*, and *Mantidactylus*). The positive occurrences seem to be associated with mid to high elevation sites, which is consistent with the climatic suitability expected for *Bd*^36^, and is similar to other regions of the world where *Bd* prevalence and *Bd*-associated declines are greater in high elevation regions^8^,^37^,^38^,^39^.

*Bd* was first detected in Madagascar in 2010 in Makay at low prevalence and intensity. In 2011 *Bd*'s presence was confirmed although with lower prevalence. Makay is a very remote massif containing relicts of humid forest within a predominantly dry region of western Madagascar, and therefore it appears as a very unusual place for an initial introduction of *Bd* to occur. Nevertheless, several hypotheses can be proposed to explain the occurrence of *Bd* in this region. One hypothesis is that a *Bd* lineage that is endemic to Madagascar has been always present in Makay. Since no sampling was completed prior to 2010, this hypothesis cannot be excluded. Museum specimens collected previously from this region need to be tested in the future for the presence of *Bd* to better understand when *Bd* arrived in the Makay region, as was done in Central America to track *Bd*'s movement through the region^40^. Alternatively, it is possible that *Bd* in Makay was introduced recently, potentially as a consequence of increased tourist activity.

At Ankarafantsika, Ankaratra, and Antoetra there were surveys yielding negative results completed within 1–2 years of the first detection of *Bd* which may suggest a recent arrival of *Bd* at these locations. In Ranomafana, surveys were only conducted in 2006 and 2007, and then in 2013 and 2014; therefore, it is difficult to make any inference about the time of arrival of *Bd* in this region. Importantly, Ranomafana National Park is one of the areas that is most visited by tourists^41^ and scientific researchers and thus may carry a greater risk of pathogen introduction. Interestingly, three individuals out of 565 (one of *Scaphiopyrne spinosa, Heterixalus betsileo*, and *H. alboguttatus*) imported to the US from Madagascar in February 2012 for the pet trade tested positive for *Bd*^33^. It is impossible to know whether these individuals were infected in the wild or as a result of contamination during shipment and transport; however, these individuals likely originated not far from Ranomafana where all three species are known to occur in sympatry^42^. Therefore, the likely origin of these specimens seems to parallel the positive occurrences seen in Ranomafana in 2013.

The use of lineage specific qPCR shows that at least some of the *Bd* detected in Madagascar is a *Bd*GPL-like lineage. The *Bd*GPL lineage occurs on every continent^43^, is associated with all of the known epizootics that have occurred, is spatially emerging on a worldwide-scale, and in experimental settings is more virulent than other lineages^44^. If it is further confirmed that the *Bd* in Madagascar is the true *Bd*GPL then it was likely introduced and may be highly virulent.

If *Bd* was introduced in Madagascar it is important to understand the route and timing of introduction. In 2003 crayfish, *Procambarus* sp., were introduced with road construction equipment from outside Madagascar near the capital, Antananarivo^45^. These organisms could be a potential source for *Bd* introduction as crayfish can be an alternative host for the pathogen^46^. However, invasive as well as native crayfish were sampled in January and February of 2014 and no *Bd* was detected. This result, along with data showing that the distribution of the invasive crayfish does not overlap with the occurrences of *Bd*, suggests that crayfish are most likely not responsible for introducing *Bd* in Madagascar. Recently it has also been documented that an alien toad species (*Duttaphrynus melanostictus*) has locally invaded eastern Madagascar^47^. If the origin of these invaders was a *Bd*-positive area of the world, then these amphibians could be *Bd*-carriers and may have subsequently introduced *Bd* to Madagascar; however, so far this species has never been recorded as a carrier of the pathogen^48^. Other possible routes of introduction include bird feathers or moist soil^49^, accidental human-assisted transport, trade of wildlife, aquarium fish and plants^50^, or machinery of foreign companies transported from diseased regions of the world^27^.

Although it is not always the case, in a scenario of recent arrival of a virulent *Bd* lineage to Madagascar, one may expect negative host effects on the frog species, similar to those seen in other tropical regions^5^,^10^. During the conducted surveys, no individuals exhibited signs of clinical chytridiomycosis, and up to now (January 2015) no mortality events associated to *Bd* occurrence have been reported in Madagascar. Based on the intensity of recent herpetological and biological fieldwork throughout Madagascar, it thus seems unlikely that amphibian mass-mortality events have occurred widely on the island. It is possible that Malagasy amphibians are in some way pre-adapted to be resistant and/or tolerant to the *Bd* lineage present in Madagascar. This concept of preadaptation of Malagasy frogs needs to be thoroughly investigated before any conclusions can be drawn. Research on the defensive function of the adaptive immunity^51^, innate immunity (AMPs)^52^ and cutaneous microbial communities^53^ can develop a better understanding of the resistance, tolerance and susceptibility of Malagasy amphibians to *Bd*.

Preliminary exposure trials showed that Malagasy amphibians can become infected with *Bd* (Supplementary Data); however, these trials do not allow for any inference about these species' susceptibility to chytridiomycosis. These data must be interpreted with caution as these trials were conducted for a short duration of time, with small sample sizes and individuals of each species were co-housed within their respective treatment.

Additional evidence for *ex situ* infectivity and susceptibility of Malagasy frogs come from a breeding facility in Tokyo where individuals of *Plethodontohyla tuberata* were found to be infected with *Bd*^54^, and from a chytridiomycosis outbreak in a zoo with high mortality of *Dyscophus* *antongilii*^55^. In spite of some Malagasy species being able to acquire infection, continued studies are needed to fully understand how Malagasy frogs would respond to the *Bd* lineage(s) identified in Madagascar as well as other genotypes that could arrive in the future.

The current lack of detection of negative effects in the wild populations may suggest that the strain of *Bd* in Madagascar is hypovirulent, as *Bd* strains are known to vary in virulence^44^. Furthermore, we cannot rule out the long-term presence of an endemic Madagascar specific genotype/lineage of *Bd* that has evaded detection due to timing of sampling and/or methodologies or that it has recently increased in prevalence as a result of shifting environmental factors. With the current data it is not possible to discriminate between the opposing hypotheses that the detected *Bd* is introduced or endemic. Importantly, these hypotheses are not mutually exclusive. It is indeed possible that the high prevalence documented in August 2013 at Ranomafana and Ankaratra was an emergence event of an introduced genotype/lineage while the detection of *Bd* at Ankaratra in 2012, Ankarafantsika, Antoetra, and particularly in Makay at lower levels was the detection of an endemic strain or lineage. Alternatively, the highly divergent prevalence recorded in Ankaratra from different years could be the result of an hybridization event between endemic and introduced strains (similar to what is reported for *Bd*Brazil x *Bd*GPL^56^), while the higher prevalence at Ranomafana in 2013 (versus the lack of detection of *Bd* in 2007) could be a result of a native *Bd* that through recombination became more virulent^44^. Continued research is needed to discern these hypotheses.

Detection of *Bd* in Madagascar appears to vary with seasons. For example, in Ankaratra at the site called Tavolotara, *Bd* was first detected in August 2012. Subsequently in August 2013, this site was again positive, with 100% prevalence; however, in December of 2013, no individuals were found positive. August is dry and cooler whereas December/January corresponds to the wet, warmer season in Madagascar. A similar trend of higher prevalence in the dry season and lower prevalence in the wet season was observed in Ankaratra at the Ambohimirandrana site as well as in Ranomafana at Vatoharanana. The same species that were previously found positive for *Bd* were resampled in the subsequent surveys, therefore, these differences in prevalence are likely not associated with the identity of the species sampled. In other regions of the world, including central America and Australia, *Bd* has been found to show dramatic seasonal trends, with higher prevalence occurring in the dryer and cooler season because cooler temperatures are more suitable for *Bd*^57^. In addition to temperature suitability, it is also possible that the seasonal dynamics of host microbial communities^53^ as well as environmental microbial and planktonic communities^58^ may be playing a role in the *Bd* dynamics observed in Madagascar.

It is important to note that different sample storage, extraction, and detection methods have been used throughout the sampling events in Madagascar (Supplementary Table 1). Under the National Monitoring Plan, the protocol proposed to test the collected samples was a simple salt extraction followed by the traditional PCR assay^59^. Although more sensitive diagnostic assays were available^60^, the intent was to have this project running continuously in Madagascar where a molecular lab equipped with a traditional PCR platform was available. The above stated methodologies were used in the first two sampling events of the NMP, although this testing (when possible) was complemented with a qPCR assay. We acknowledge that variation in methodologies may confound time and season with detection method and could in part compromise the conclusions of the recent detection of *Bd* and seasonality trends. This does not compromise one of our main conclusions - that *Bd* has been detected in wild amphibians in Madagascar. It does, however, stress the importance of standardizing protocols for future investigations^61^. We suggest the following methodologies: 1) swabbing should be done with fine tip swabs and samples should be stored dry in cool temperatures, as recommended in Hyatt et al.^62^, 2) for extraction, Prepman should be used (although when time and money are not a constraint, Qiagen DNeasy extraction kits should be used as this is the best balance of efficiency and removal of PCR inhibition^63^), 3) for the detection assay we suggest the use of qPCR with BSA which is currently the most sensitive detection assay for *Bd*^60^,^62^. In addition, it will be important to standardize the reference strain of *Bd* used in different laboratories or to use cloned DNA fragment standards to allow accurate comparisons.

In light of *Bd*'s presence in Madagascar it is imperative to ensure continuous monitoring across the country, especially at the sites already monitored under the NMP, at sites that have tested positive for Bd, and at mid-high altitude sites, where the pathogen is more likely to be present^64^. This practice will develop a better understanding of *Bd* trends and dynamics in Madagascar enabling an effective response to an emerging threat. To minimize the spread of *Bd*, all researchers must adopt strict hygiene protocols^31^,^65^. While we do not know the virulence and real impacts of the *Bd* present in Madagascar, it is crucial to apply a precautionary principle and ensure surveillance of the frog populations to facilitate early detection of declines due to the importance of Malagasy amphibian species to global amphibian diversity. A further research priority must be to isolate the Malagasy *Bd* lineage(s) so that experimental approaches can be used to determine its virulence and evolutionary history.

In coordination with the Malagasy authorities, researchers and conservationists must prepare stakeholders for an effective response to a chytridiomycosis outbreak by the development and implementation of disease mitigation strategies^66^. In 2010, the first *in-situ* amphibian breeding facility was established in Andasibe, Madagascar, which can serve as a model for other captive assurance breeding centres, such as the coming breeding centre in Ivoloina Zoological Park^67^. These facilities may become vital resources for housing and preserving species if *Bd*-associated declines are documented^30^. Furthermore, probiotic therapy for amphibians is a promising disease mitigation strategy and can provide a potential mechanism to combat *Bd* in Madagascar^53^. In vitro assays of Malagasy amphibian skin bacteria against *Bd* have shown that some of the collected bacteria can strongly inhibit *Bd* growth. Similarly, recent discoveries^58^ in other regions of the world suggest that understanding and characterizing the microorganism communities of freshwater systems where *Bd* is present (or absent) may be important since these organisms could be part of a natural integrated strategy to reduce the spread of *Bd* and its infection potential.

We have documented the presence of *Bd* in wild amphibian populations in Madagascar; however, evidence for clinical signs of chytridiomycosis is so far lacking. The rarity of pre-2013 positives and the low intensity values found, in contrast with the high prevalence and intensity values found recently suggests either an emergence event, or a high degree of seasonality leading us to misdiagnose infection status in previous years in Madagascar. Continued research to fully understand the distribution, origin, type and virulence of the *Bd* lineage(s) present in Madagascar is essential and increased capacity to develop and implement conservation strategies are imperative for the successful conservation of Malagasy amphibians.

## Methods

### Site information

See Supplementary Table 1 for a list of the sites across Madagascar that were sampled for *Bd* (Figure 1). Sampling effort has been distributed across all bioclimatic regions of Madagascar (Figure 2, Supplementary Table 1).

### Frog capture and sampling methods

NMP surveys were coordinated by the CEC: three focal species were selected and sampled at each of the eight monitored sites^31^. Surveys were completed in 2011–2013 by selected conservation organisations^31^. The last year of sampling for the first 3-years period of the NMP was recently concluded. For independent surveys undertaken outside the NMP, frogs were captured opportunistically during day and night searches. For all surveys, each individual was swabbed on its ventral surface of its abdomen, hind limbs and feet 5–10 times with a sterile fine-tip swab. Swabs collected under the NMP and independent surveys completed in 2007–2008 were immediately stored in unique vials with 96% ethanol. For independent surveys completed in 2013–2014 swabs were stored dry in 1.5 ml vials on ice until access to a freezer was available. In all conducted surveys, individuals were collected with newly gloved hands and placed in separate bags until processing to prevent cross contamination. Frogs were immediately released at the site of capture after sampling. In order to avert potential cross-contamination, hygiene procedures, including washing boots and all reusable sampling equipment with a 10% bleach solution were performed between sites.

### DNA extraction and PCR detection analysis

Different DNA extraction methods have been used for the collected swab samples, including PrepMan Ultra Reagent Protocol as described by Boyle et al.^60^, standard salt extraction as described in Weldon et al.^31^ or a modification of this method as described in Bandi et al.^68^, Mobio PowerSoil DNA Isolation Kit (MoBio, Carlsbad, CA) as described by Costello et al.^69^ and 5 Prime Archive Pure Kit (Supplementary Table 1). For tissue samples, Qiagen DNeasy Blood and Tissue Kits (Qiagen, Valencia, CA) were used according to the manufacturer's protocol for animal tissues. DNA extracts were stored at −20, −25 or −80°C until downstream processing.

Tissue and swabs samples were assessed for the presence of *Bd* using both traditional polymerase chain reaction (PCR) and quantitative real-time polymerase chain reaction (qPCR). Samples from Andasibe (2011), Ankarafantsika (2011), Ankaratra (Oct 2010), Ankaratra (May 2012), Antoetra (2011–2012), Itremo (2008), Mandena (2011), Masoala (2012), and Toamasina (2011) were all tested using traditional PCR. All other samples were tested with qPCR or with both PCR and qPCR (Supplementary Table 1). Traditional PCR was performed according to Annis et al.^59^ while qPCR analyses were performed in accordance to the protocol outlined by Boyle et al.^60^. All extracts from PrepMan and salt extraction procedures were diluted 1:10 prior to PCR/qPCR analysis while all other extracts remained undiluted for testing. For qPCR, standards of known zoospore concentrations (made from a GPL isolate-*Bd* JEL 423 provided by Joyce Longcore- University of Maine) and negative controls were included in each qPCR plate. Each sample was run in duplicate qPCR reactions and single replicate positives were rerun. Samples were considered positive when amplification occurred in two qPCR reactions and the GEs quantity was greater than 0.1 GE (genome equivalents: reported as mean value for each sample). We used these values as an index of the intensity of an individual's infection. For a subset of samples from each lab completing qPCR analyses exogenous internal positive controls were included as described by Hyatt et al.^62^ to test for PCR inhibition. We found no evidence of PCR inhibition.

It is important to note, for the qPCR results, that different strains of *Bd* have different copy numbers of the ITS1-5.8S DNA fragment^70^, in part due to variable chromosomal copy numbers among strains and within strains^44^,^71^. Therefore, comparisons of the *Bd* infection intensities performed by different teams should be made with caution. For the same reasons, infection intensity should be interpreted with caution, as it is not yet known what type of *Bd* is present in Madagascar. Importantly, these issues are less of a concern for presence-absence data.

### Lineage-specific qPCR

Singleplex quantitative PCR reactions utilizing Taqman MGB probes were used to discriminate single nucleotide polymorphisms in the *Bd* mitochondrial genome that are diagnostic for three major lineages of *Bd*: *Bd*GPL, *Bd*CAPE and *Bd*CH^44^,^72^,^73^. qPCR conditions were adapted from the conditions described by Boyle et al.^60^, differing at the annealing step where the temperature was raised from 60°C to 62°C. These methods were performed on the samples collected in 2010 and 2011 at Makay, a set of samples from 5 locations sampled in 2012 under the NMP, and a subset of samples from August 2013 collected by Bletz and colleagues in 2013 and 2014 (Supplementary Table 1).

### Crayfish sampling

In 2013, crayfish were found to be an alternative host for *Bd*^46^. The recent introduction of an invasive crayfish in Madagascar raised concerns about the role of the species as a possible source of *Bd* introduction and therefore, we decided to include crayfish sampling in the following year to investigate this hypothesis. In January 2014, crayfish in Antananarivo (n = 55), Mandraka (n = 10), and Ranomafana (n = 11) were surveyed for *Bd*. In Antananarivo and Mandraka, individuals of the introduced invasive species *Procambarus* spp. were collected while in Ranomafana individuals of the native species *Astacoides* sp. were collected. Crayfish were euthanized via freezing and the gastrointestinal (GI) tract was dissected from each individual. The dissected GI tracts were stored in EtOH until laboratory processing. The intestinal tissue was cleaned using sterilized scissors and forceps to remove waste and debris. DNA was extracted from intestinal tissue using Qiagen DNeasy Blood and Tissue Kit, according to the manufacturer's protocol for animal tissues and qPCR as described above was used to test for the presence of *Bd*.

### Exposure trial methods

Preliminary exposure trials were carried out on six species of Malagasy frogs (*Boophis madagascariensis*, *B. viridis, Heterixalus betsileo, Guibemantis liber, Mantidactylus betsileanus*, and *Ptychadena mascareniensis*), at the North-West University (NWU) in Potchefstroom, South Africa. All experimental methods were carried out in accordance with the approved guidelines and protocols under the ethics permit no. NWU-00013-10-S4 issued by the NWU Research Ethics Committee. After a 12-day acclimation period, individuals were assigned either to a control treatment or exposed to a 5-day regiment of *Bd*GPL (strain MG04 isolated from *Amietia fuscigula*, Western Cape, South Africa). Frogs were swabbed before treatment and after six, 15 and 20 days, and samples analysed with qPCR. See Supplementary Materials for more details on methods.

### Statistical Analysis

Given the *ad-hoc* nature of the sampling design, no formal statistical analyses were performed, which avoids giving false confidence to preliminary findings.

## Author Contributions

M.C.B., G.M.R., A.C., M.V., M.F., R.N.H. and F.A. conceived and designed the study. M.C.B., G.M.R., A.C., E.A.C., N.R., F.R., M.V., C.W., D.E., C.R., R.N.H. and F.A. performed sampling, and M.C.B., G.M.R., A.C., E.A.C., D.S.S., C.W. and M.C.F. performed *Bd* screening. C.W. and L.R. performed exposure experiments. M.C.B., G.M.R. and A.C. analysed the data and wrote the paper. All authors reviewed the manuscript.

## Supplementary Material

Supplementary Information Supplementary Information

## Figures and Tables

**Table 1:** *Bd* detection at each location over time, within each bioclimatic region and elevational classifications. Sampling years from 2010–2014 are divided into 4-month blocks: January-April (J-A), May-August (M-A) and September-December (S-D). *Bd*-positive and *Bd*-negative occurrences are indicated by “+” and “−” respectively. Results from multiple sites at a given location are included. If *Bd* occurred at least one site within the location then a “+” is presented. Grey fill indicates time before the first sampling

Bioclimatic region	Elevation	Location	2005	2006	2007	2008	2009	2010			2011			2012			2013			2014
								J-A	M-A	S-D	J-A	M-A	S-D	J-A	M-A	S-D	J-A	M-A	S-D	J-A
Subhumid	High	Antananarivo		−																
		Ambohintantely	−																	−
		Itremo				−														
		Antoetra											−	−		+				+
		Montagne d' Ambre				−														
Humid	Mid	An'Ala		−																−
		Andasibe	−	−									−			−		−		−
		Fierenana																		−
		Ranomafana		−	−													+		+
		Torotorofotsy																−		−
	Low	Manakara			−															
		Mandena											−		−					
		Manombo			−															
		Masoala		−										−	−		−	−	−	
		Toamasina								−			−		−		−	−		
Montane	High	Andringitra			−															
		Ankaratra	−	−						−					−/+			−/+	+	
Subarid	Low	Ifaty			−															
		Menabe												−			−			
		Toliara			−															
	Mid	Isalo			−															
Dry	Mid	Ankarafantsika			−								−	+			−			
		Makay								+		+							−	

**Table 2:** Frog species yielding positive *Bd* detection results for each location where *Bd* has been detected. Candidate species names after Perl et al.^19^

Year	Location	Species
2010	Makay	*Mantidactylus* sp. Ca14
2011	Makay	*Ptychadena mascareniensis*
2012	Ankarafantsika	Unknown
	Antoetra	*Mantidactylus* sp. Ca48
	Ankaratra	*Mantidactylus pauliani*
2013	Ankaratra	*Boophis williamsi*; *B. goudoti*; *B. ankaratra*; *Mantidactylus pauliani*; *M.* sp. Ca19; *Ptychadena mascareniensis*; *Heterixalus betsileo*
	Ranomafana	*Boophis idae*; *B. madagascariensis*; *B. quasiboehmei*; *Gephyromantis asper; Mantidactylus betsileanus*; *M. majori*; *Platypelis pollicaris*
2014	Antoetra	*Mantella cowani*
	Ranomafana	*Boophis reticulatus*

**Table 3:** Zoospore intensities for lineage specific qPCR for three *Bd* lineages (*Bd*GPL, *Bd*CAPE, and *Bd*CH) at different locations across Madagascar. Detection(+)/No Detection (−) of *Bd* for standard ITS qPCR is also provided. NT indicates that the lineage probe was not tested for that sample. *Note: in August 2013 samples collected from Ranomafana and Ankaratra that showed no lineage probe amplification are presented together and the number of samples are provided in parentheses*

			Probe			
Location	Year	Type	*Bd* ITS rDNA	*Bd*GPL mtDNA	*Bd*CAPE mtDNA	*Bd*CH mtDNA
Makay	2010	Individual	+	NT	−	NT
Makay	2011	Individual	+	NT	−	NT
Menabe	Jan 2012	Pooled	−	0	0	0
Andasibe	Sept 2012	Pooled	−	0	0	0
Ankaratra	Aug 2012	Pooled	+	0	0	0
Ankarafantsika	Mar 2012	Pooled	+	4	0	0
Antoetra	Oct 2012	Pooled	+	17	0	0
Ranomafana	Aug 2013	Individual	+	9	0	0
Ranomafana	Aug 2013	Individual	+	1	0	0
Ranomafana	Aug 2013	Individual	+	1	0	0
Ranomafana	Aug 2013	Individuals (12)	+	0	0	0
Ankaratra	Aug 2013	Individual	+	2	0	0
Ankaratra	Aug 2013	Individual	+	8	0	0
Ankaratra	Aug 2013	Individuals (11)	+	0	0	0
Ankaratra	Aug 2013	Individual	+	10	0	0
Ankaratra	Aug 2013	Individuals (5)	+	0	0	0
