# Supplementary material for: Widespread presence of the pathogenic fungus Batrachochytrium dendrobatidis in wild amphibian communities in Madagascar
Source: Sci Rep. 2015 Feb 26;5:8633. doi: 10.1038/srep08633 (PMC4341422; doi:10.1038/srep08633)
Supplement: Supplementary Information [file srep08633-s1.pdf]

**Bletz, Rosa et al.: Widespread presence of the pathogenic fungus Batrachochytrium dendrobatidis in wild amphibian communities in Madagascar**

Molly C. Bletz[1,2*†], Gonçalo M. Rosa[3,4,5*†] , Franco Andreone [6,7*], Elodie A. Courtois[8,9], Dirk S. Schmeller,[10,11], Nirhy H. C. Rabibisoa[7,12], Falitiana C. E. Rabemananjara,[13], Liliane Raharivololoniaina[13], Miguel Vences[2], Ché Weldon[14], Devin Edmonds[15], Christopher J. Raxworthy[16], Reid N. Harris[1], Matthew C. Fisher[17], Angelica Crottini[18]

**Supplementary Table 1:** Detailed information for each sampling event, including region, location, site name, GPS coordinates, year and month sampled, Bd presence, zoospore equivalent range, number of Bd positive samples, prevalence, sample size, number of species sampled, sample type, extraction and detection method, and field and laboratory team. For sample type, (d) indicates swabs were stored dry and (e) indicates swabs were stored in ethanol. For extraction method the following abbreviations are used: PrepMan Ultra (PMU), Salt Extraction (SE), Mobio PowerSoil (MBPS), Qiagen DNeasy (QD), and 5 Prime Archive Pure Kit (5PAP). For Laboratory team the following abbreviations are used: San Francisco State University (SF State U.), Università di Milano-Bicocca (UMB), Imperial College (Imp C.), Institute of Zoology, Zoological Society of London (ZSL), James Madison University (JMU), Centre National de la Recherche Scientifique (CNRS), San Diego Zoo Amphibian Disease Laboratory (SDZ), NorthWest University- South Africa, (NWU), and National Zoological Gardens of South Africa (NZG SA). * indicates which samples were used in lineage specific qPCR analysis. ♦ indicates sites where the GPS coordinates are only approximate references as specific GPS data was not available.

| Region | Location | Site | Latitude | Longitude | Year | Month | Season | Elevation | Bd presence | GE range | Prevalence | Sample size | # species | Sample type | Extraction | Detection | Field team | Lab team | Publication |
|---|---|---|---|---|---|---|---|---|---|---|---|---|---|---|---|---|---|---|---|
| Dry | Ankarafantsika | Ampijoroa ♦ | -16.233052 | 46.475379 | 2007 | Jan-Feb | Wet | Mid | NEG | | 0 | 49 | 8 | swab (d) | PMU | qPCR | Weldon & Vences Team | SF State U. | Vredenburg et al. 2012 |
| Dry | Ankarafantsika | Andranofasika | -16.29889 | 46.813 | 2011 | Nov | Wet | Mid | NEG | | 0 | 50 | 3 | swab (e) | SE | PCR | NMP-Chytrid Emergency Cell | UMB | |
| Dry | Ankarafantsika* | Andranofasika | -16.29889 | 46.813 | 2012 | Mar | Dry | Mid | POS | 2 | NA | 50 | UNK | swab (e) | SE | PCR & qPCR | NMP-Chytrid Emergency Cell | UMB/Imp. C | |
| Dry | Ankarafantsika | Andranofasika | -16.29889 | 46.813 | 2013 | Jan | Wet | Mid | NEG | | 0 | 50 | 4 | swab (e) | PMU | qPCR | NMP-Chytrid Emergency Cell | ZSL | |
| Dry | Makay | Antsoha, Androtsy &Ankilytsoky ♦ | -21.59598 (Antsoha) | 45.11325 (Antsoha) | 2011 | Jan | Wet | High | NEG | | 0 | 18 | 13 | tissue | SE | PCR & Nested PCR | Raxworthy Team | UMB | |
| Dry | Makay | Camp Palmiers | -21.8816 | 45.413 | 2010 | Dec | Wet | High | NEG | | 0 | 28 | 1 | swab | PMU | PCR & qPCR | Courtois Team | CNRS/UMB/Imp. C | Rabemananjara et al. 2011 |
| Dry | Makay | Camp Lac | -21.94333 | 46.1016 | 2010 | Nov | Wet | High | NEG | | 0 | 37 | 3 | swab | PMU | PCR & qPCR | Courtois Team | CNRS/UMB/Imp. C | Rabemananjara et al. 2011 |
| Dry | Makay | Canyon Chauve Souris | -21.59 | 45.82833 | 2010 | Dec | Wet | High | NEG | | 0 | 22 | 2 | swab | PMU | PCR & qPCR | Courtois Team | CNRS/UMB/Imp. C | Rabemananjara et al. 2011 |
| Dry | Makay | Canyon Orage | -22.0933 | 45.4966 | 2010 | Dec | Wet | High | NEG | | 0 | 38 | 1 | swab | PMU | PCR & qPCR | Courtois Team | CNRS/UMB/Imp. C | Rabemananjara et al. 2011 |
| Dry | Makay | Andranovinily | -22.12 | 46.01166 | 2010 | Dec | Wet | High | POS | 0.157-.273 | 8.11 | 37 | 1 | swab | PMU | PCR & qPCR | Courtois Team | CNRS/UMB/Imp. C | Rabemananjara et al. 2011 |
| Dry | Makay | Andranovinily | -22.12 | 46.01166 | 2011 | Aug | Dry | High | NEG | | 0 | 87 | 6 | swab | PMU | qPCR | Andreone/Weldon Teams | Imp. C | |
| Dry | Makay | Andranovinily | -22.12 | 46.01166 | 2013 | Oct | Wet | High | NEG | | 0 | 40 | 3 | tissue | PMU | qPCR | Andreone Team | ZSL | |
| Dry | Makay | Anosilahy/Lac Sacré | -21.354 | 45.147 | 2011 | Aug | Dry | High | NEG | | 0 | 67 | 4 | swab | PMU | qPCR | Andreone/Weldon Teams | Imp. C | |
| Dry | Makay | Beronono | -21.3614 | 45.24798 | 2011 | Aug | Dry | High | NEG | | 0 | 63 | 2 | swab | PMU | qPCR | Andreone/Weldon Teams | Imp. C | |
| Dry | Makay | Beronono | -21.3614 | 45.24798 | 2013 | Oct | Wet | High | NEG | | 0 | 40 | 1 | tissue | PMU | qPCR | Andreone Team | ZSL | |
| Dry | Makay | Beroroha | -21.67921 | 45.16851 | 2011 | Aug | Dry | High | POS | 0.239 | 1.15 | 87 | 2 | swab | PMU | qPCR | Andreone/Weldon Teams | Imp. C | |
| Dry | Makay | Beroroha | -21.67921 | 45.16851 | 2013 | Oct | Wet | High | NEG | | 0 | 122 | 2 | tissue | PMU | qPCR | Andreone Team | ZSL | |
| Dry | Makay | Saririaky | -21.35785 | 45.17626 | 2011 | Aug | Dry | High | NEG | | 0 | 59 | 4 | swab | PMU | qPCR | Andreone/Weldon Teams | Imp. C | |
| Dry | Makay | Saririaky | -21.35785 | 45.17626 | 2013 | Oct | Wet | High | NEG | | 0 | 27 | 1 | tissue | PMU | qPCR | Andreone Team | ZSL | |
| Humid | An'Ala | Vohimana Reserve | -18.920857 | 48.514697 | 2006 | Feb | Wet | Mid | NEG | | 0 | 178 | UNK | histology | NA | NA | Weldon & Vences Team | NWU | Weldon et al. 2008 |
| Humid | An'Ala | Vohimana Reserve | -18.920857 | 48.514697 | 2006 | Feb | Wet | Mid | NEG | | 0 | 27 | 13 | swab (d) | PMU | qPCR | Weldon & Vences Team | SF State U. | Vredenburg et al. 2012 |
| Humid | An'Ala | Vohimana Reserve | -18.920857 | 48.514697 | 2014 | Feb | Wet | Mid | NEG | | 0 | 31 | 12 | swab (d) | MBPS | qPCR | Bletz Team | JMU | |
| Humid | Andasibe | Analamazaotra & Andasibe village | -18.92711 -18.92360 | 48.41569 48.42202 | 2005 | Jan | Wet | Mid | NEG | | 0 | 28 | UNK | histology | NA | NA | Weldon & Vences Team | NWU | Weldon et al. 2008 |
| Humid | Andasibe | Analamazaotra & Andasibe village | -18.92711 -18.92360 | 48.41569 48.42202 | 2006 | Feb | Wet | Mid | NEG | | 0 | 42 | UNK | histology | NA | NA | Weldon & Vences Team | NWU | Weldon et al. 2008 |
| Humid | Andasibe | Analamazaotra & Andasibe village | -18.92711 -18.92360 | 48.41569 48.42202 | 2006 | Feb | Wet | Mid | NEG | | 0 | 25 | 14 | swab (d) | PMU | qPCR | Weldon & Vences Team | SF State U. | Vredenburg et al. 2012 |
| Humid | Andasibe | Analamazaotra & Andasibe village | -18.92711 -18.92360 | 48.41569 48.42202 | 2011 | Oct | Wet | Mid | NEG | | 0 | 50 | 2 | swab (e) | SE | PCR | NMP-Chytrid Emergency Cell | UMB | |
| Humid | Andasibe* | Analamazaotra & Andasibe village | -18.92711 -18.92360 | 48.41569 48.42202 | 2012 | Sept | Dry | Mid | NEG | | 0 | 50 | 2 | swab (e) | SE | PCR & qPCR | NMP-Chytrid Emergency Cell | UMB/Imp. C | |
| Humid | Andasibe | Analamazaotra & Andasibe village | -18.92711 -18.92360 | 48.41569 48.42202 | 2013 | Feb | Wet | Mid | NEG | | 0 | 60 | 2 | swab (e) | PMU | qPCR | NMP-Chytrid Emergency Cell | ZSL | |
| Humid | Andasibe | Analamazaotra & Andasibe village | -18.92711 -18.92360 | 48.41569 48.42202 | 2013 | June | Dry | Mid | NEG | | 0 | 60 | 2 | swab (e) | PMU | qPCR | NMP-Chytrid Emergency Cell | ZSL | |
| Humid | Andasibe | MMA & Analamazaotra | -18.92654 -18.92711 | 48.41775 48.41569 | 2013 | Aug | Dry | Mid | NEG | | 0 | 26 | 7 | swab (d) | MBPS | qPCR | Bletz Team | JMU/SDZ | |
| Humid | Andasibe | MMA & Mitsinjo | -18.92654 -18.93283 | 48.41775 48.41312 | 2014 | Feb | Wet | Mid | NEG | | 0 | 15 | 7 | swab (d) | MBPS | qPCR | Bletz Team | JMU | |
| Humid | Fierenana | Beravotavo | -18.60177 | 48.43417 | 2014 | Jan | Wet | Mid | NEG | | 0 | 29 | 8 | swab (d) | MBPS | qPCR | Bletz Team | JMU | |
| Humid | Manakara | Manakara Town ♦ | -22.151328 | 48.012247 | 2007 | Feb | Wet | Low | NEG | | 0 | 16 | 2 | swab (e) | PMU | qPCR | Vences Team | ZSL | |
| Humid | Manombo | Manombo Special Reserve ♦ | -23.020645 | 47.724607 | 2007 | Feb | Wet | Low | NEG | | 0 | 34 | 16 | swab (e) | PMU | qPCR | Vences Team | ZSL | |
| Humid | Mandena | Zone de conservation & mining site QMM ♦ | -24.965945 | 46.880293 | 2011 | Oct | Wet | Low | NEG | | 0 | 50 | 2 | swab (e) | SE | PCR | NMP-Chytrid Emergency Cell | UMB | |
| Humid | Mandena | Zone de conservation & mining site QMM ♦ | -24.965945 | 46.880293 | 2013 | July | Dry | Low | NEG | | 0 | 60 | 3 | swab (e) | PMU | qPCR | NMP-Chytrid Emergency Cell | Imp. C | |
| Humid | Masoala | Farankaraina | -15.4333 | 49.8409 | 2012 | Mar | Wet | Low | NEG | | 0 | 50 | 2 | swab (e) | SE | PCR | NMP-Chytrid Emergency Cell | UMB | |
| Humid | Masoala | Farankaraina | -15.4333 | 49.8409 | 2012 | July | Dry | Low | NEG | | 0 | 50 | 2 | swab (e) | SE | PCR | NMP-Chytrid Emergency Cell | UMB | |
| Humid | Masoala | Farankaraina | -15.4333 | 49.8409 | 2013 | Sept | Dry | Low | NEG | | 0 | 89 | 31 | swab (d) | MBPS | qPCR | Bletz Team | JMU/SDZ | |
| Humid | Masoala | Farankaraina | -15.4333 | 49.8409 | 2013 | Mar | Wet | Low | NEG | | 0 | 50 | 2 | swab (e) | PMU | qPCR | NMP-Chytrid Emergency Cell | ZSL | |
| Humid | Masoala | Farankaraina | -15.4333 | 49.8409 | 2013 | Jul | Dry | Low | NEG | | 0 | 50 | 2 | swab (e) | PMU | qPCR | NMP-Chytrid Emergency Cell | ZSL | |
| Humid | Masoala | Maroantsetra | -15.43329 | 49.74187 | 2006 | Feb | Wet | Low | NEG | | 0 | 43 | UNK | histology | NA | NA | Weldon & Vences Team | NWU | Weldon et al. 2008 |
| Humid | Masoala | Maroantsetra | -15.43329 | 49.74187 | 2013 | Sept | Dry | Low | NEG | | 0 | 5 | 1 | swab (d) | MBPS | qPCR | Bletz Team | JMU/SDZ | |
| Humid | Masoala | Nosy Mangebe | -15.496685 | 49.76197 | 2013 | Sept | Dry | Low | NEG | | 0 | 46 | 12 | swab (d) | MBPS | qPCR | Bletz Team | JMU/SDZ | |
| Humid | Ranomafana | Multiple sites ♦ | -21.256363 | 47.420876 | 2006 | Feb | Wet | Mid | NEG | | 0 | 115 | UNK | histology | NA | NA | Weldon & Vences Team | NWU | Weldon et al. 2008 |
| Humid | Ranomafana | Multiple sites ♦ | -21.256363 | 47.420876 | 2006 | Feb | Wet | Mid | NEG | | 0 | 23 | 11 | swab (d) | PMU | qPCR | Weldon & Vences Team | SF State U. | Vredenburg et al. 2012 |
| Humid | Ranomafana | Ambatolahy | -21.24599 | 47.42489 | 2007 | Jan-Feb | Wet | Mid | NEG | | 0 | 24 | 11 | swab (d) | PMU | qPCR | Weldon & Vences Team | SF State U. | Vredenburg et al. 2012 |
| Humid | Ranomafana | Ambatoladimy | -21.25034 | 47.42057 | 2014 | Jan | Wet | Mid | NEG | | 0 | 23 | 8 | swab (d) | MBPS | qPCR | Bletz Team | JMU | |
| Humid | Ranomafana* | Vatoharanana* | -21.28884 | 47.42972 | 2013 | Aug | Dry | Mid | POS | 16.7-145.8 | 50 | 30 | 9 | swab (d) | MBPS | qPCR | Bletz Team | JMU/SDZ | |
| Humid | Ranomafana | Vatoharanana | -21.28884 | 47.42972 | 2014 | Jan | Wet | Mid | NEG | | 0 | 87 | 27 | swab (d) | MBPS | qPCR | Bletz Team | JMU | |
| Humid | Ranomafana | Vohiparara | -21.23565 | 47.39752 | 2006 | Feb | Wet | Mid | NEG | | 0 | 14 | 6 | swab (d) | PMU | qPCR | Weldon & Vences Team | SF State U. | Vredenburg et al. 2012 |
| Humid | Ranomafana | Vohiparara | -21.23565 | 47.39752 | 2014 | Jan | Wet | Mid | NEG | | 0 | 46 | 16 | swab (d) | MBPS | qPCR | Bletz Team | JMU | |
| Humid | Ranomafana | Maharira | -21.159117 | 47.40245 | 2014 | Jan | Wet | Mid | NEG | | 0 | 24 | 7 | swab (d) | QD | qPCR | Hutter Team | JMU | |
| Humid | Ranomafana | Ranomafanakely | -21.24866 | 47.37186 | 2014 | Jan | Wet | Mid | NEG | | 0 | 8 | 5 | swab (d) | MBPS | qPCR | Bletz Team | JMU | |
| Humid | Ranomafana | Valohoaka | -21.2971 | 47.4404 | 2014 | Jan | Wet | Mid | POS | 2.53 | 2.33 | 43 | 14 | swab (d) | QD | qPCR | Hutter Team | JMU | |
| Humid | Toamasina | Ivoloina Zool. Park | -18.05837 | 49.35797 | 2010 | Oct | Wet | Low | NEG | | 0 | 59 | 12 | swab (d) | PMU | qPCR | Andreone & Weldon Team | NZG SA | Crottini et al. 2013 |
| Humid | Toamasina | Ivoloina Zool. Park | -18.05837 | 49.35797 | 2011 | Oct | Wet | Low | NEG | | 0 | 50 | 2 | swab (e) | SE | PCR | NMP-Chytrid Emergency Cell | UMB | |
| Humid | Toamasina | Ivoloina Zool. Park | -18.05837 | 49.35797 | 2012 | July | Dry | Low | NEG | | 0 | 50 | 2 | swab (e) | PMU | qPCR | NMP-Chytrid Emergency Cell | ZSL | |
| Humid | Toamasina | Ivoloina Zool. Park | -18.05837 | 49.35797 | 2013 | Feb | Wet | Low | NEG | | 0 | 50 | 2 | swab (e) | PMU | qPCR | NMP-Chytrid Emergency Cell | ZSL | |
| Humid | Toamasina | Ivoloina Zool. Park | -18.05837 | 49.35797 | 2013 | July | Dry | Low | NEG | | 0 | 50 | 2 | swab (e) | PMU | qPCR | NMP-Chytrid Emergency Cell | ZSL | |
| Humid | Torotorofotsy | Prolemur simus Camp | -18.77085 | 48.43215 | 2013 | Sept | Dry | Mid | NEG | | 0 | 14 | 5 | swab (d) | MBPS | qPCR | Bletz Team | JMU/SDZ | |
| Humid | Torotorofotsy | Prolemur simus Camp | -18.77085 | 48.43215 | 2014 | Feb | Wet | Mid | NEG | | 0 | 36 | 14 | swab (d) | MBPS | qPCR | Bletz Team | JMU | |
| Montane | Andringitra | Multiple sites in montane environment | -22.146 | 46.947 | 2007 | Jan | Wet | High | NEG | | 0 | 48 | UNK | swab (d) | PMU | qPCR | Vences Team | ZSL | |
| Montane | Ankaratra | Multiple sites between Nosiarivo and Tsiafajavona ♦ | -19.350098 | 47.243333 | 2005 | Jan | Wet | High | NEG | | 0 | 27 | UNK | histology | NA | NA | Weldon & Vences Team | NWU | Weldon et al. 2008 |
| Montane | Ankaratra | Multiple sites between Nosiarivo and Tsiafajavona ♦ | -19.350098 | 47.243333 | 2006 | Feb | Wet | High | NEG | | 0 | 22 | UNK | histology | NA | NA | Weldon & Vences Team | NWU | Weldon et al. 2008 |
| Montane | Ankaratra | Multiple sites ♦ | -19.341897 | 47.290939 | 2006 | Feb | Wet | High | NEG | | 0 | 41 | 8 | swab (d) | PMU | qPCR | Weldon & Vences Team | SF State U. | Vredenburg et al. 2012 |
| Subhumid | Ankaratra | Ambatolampy | -19.38136 | 47.42566 | 2006 | Feb | Wet | High | NEG | | 0 | 32 | | histology | NA | NA | Weldon & Vences Team | NWU | Weldon et al. 2008 |
| Subhumid | Ankaratra | Ambatolampy | -19.38136 | 47.42566 | 2006 | Feb | Wet | High | NEG | | 0 | 21 | 2 | swab (d) | PMU | qPCR | Weldon & Vences Team | SF State U. | Vredenburg et al. 2012 |
| Subhimud | Ankaratra* | Ambatolampy* | -19.38136 | 47.42566 | 2013 | Aug | Dry | High | POS | 15.9-97.5 | 63.64 | 11 | 2 | swab (d) | MBPS | qPCR | Bletz Team | JMU/SDZ | |
| Montane | Ankaratra | Ambohimirandrana & Tavolotara[e] | -19.34079 -19.34634 | 47.27428 47.27705 | 2010 | Oct | Wet | High | NEG | | 0 | 18 | 4 | tissue | SE | PCR | Andreone Team | UMB | |
| Montane | Ankaratra | Ambohimirandrana | -19.34079 | 47.27428 | 2013 | Dec | Wet | High | POS | 0.75 | 2.86 | 35 | 2 | swab (e) / tissue | PMU/ QD | qPCR | Chytrid Emergency Cell/ Durrel/ Mitsinjo | ZSL | |
| Montane | Ankaratra* | Ambohimirandrana* | -19.34079 | 47.27428 | 2013 | Aug | Dry | High | POS | 47.3-95.15 | 100 | 7 | 2 | swab (d) | MBPS | qPCR | Bletz Team | JMU/SDZ | |
| Montane | Ankaratra | Analafohy | -19.34416 | 47.27508 | 2013 | Dec | Wet | High | NEG | | 0 | 33 | 3 | swab (e) / tissue | PMU/ QD | qPCR | Chytrid Emergency Cell/ Durrel/ Mitsinjo | ZSL | |
| Montane | Ankaratra | Andraraty | -19.36837 | 47.33161 | 2012 | May | Wet | High | NEG | | 0 | 50 | 2 | swab (e) | SE | PCR | NMP-Chytrid Emergency Cell | UMB | |
| Montane | Ankaratra | Andraraty | -19.36837 | 47.33161 | 2013 | June | Dry | High | NEG | | 0 | 30 | 1 | swab (e) | PMU | qPCR | NMP-Chytrid Emergency Cell | ZSL | |
| Montane | Ankaratra | Andraraty | -19.36837 | 47.33161 | 2013 | Dec | Wet | High | NEG | | 0 | 20 | 3 | swab (e) / tissue | PMU/ QD | qPCR | Chytrid Emergency Cell/ Durrel/ Mitsinjo | ZSL | |
| Montane | Ankaratra* | Tavolotara | -19.34634 | 47.27705 | 2012 | Aug | Dry | High | POS | 11 | NA | 50 | 1 | swab (e) | SE | PCR & qPCR | NMP-Chytrid Emergency Cell | UMB/Imp. C | |
| Montane | Ankaratra | Tavolotara & Ambatomena | -19.34634 | 47.27705 | 2013 | Feb | Wet | High | NEG | | 0 | 59 | 1 | swab (e) | PMU | qPCR | NMP-Chytrid Emergency Cell | ZSL | |
| Montane | Ankaratra* | Tavolotara* | -19.34634 | 47.27705 | 2013 | Aug | Dry | High | POS | 37.3-167.86 | 100 | 18 | 5 | swab (d) | MBPS | qPCR | Bletz Team | JMU/SDZ | |
| Montane | Ankaratra | Tavolotara | -19.34634 | 47.27705 | 2013 | Dec | Wet | High | NEG | | 0 | 22 | 4 | swab (e) / tissue | PMU/ QD | qPCR | Chytrid Emergency Cell/ Durrel/ Mitsinjo | ZSL | |
| Montane | Ankaratra | Tavolotara Lower | -19.36443 | 47.29869 | 2013 | Dec | Wet | High | NEG | | 0 | 26 | 3 | swab (e) / tissue | PMU/ QD | qPCR | Chytrid Emergency Cell/ Durrel/ Mitsinjo | ZSL | |
| Montane | Ankaratra | Tsimiaramianadahy | -19.33381 | 47.26386 | 2013 | Dec | Wet | High | NEG | | 0 | 25 | 6 | swab (e) / tissue | PMU/ QD | qPCR | Chytrid Emergency Cell/ Durrel/ Mitsinjo | ZSL | |
| Subarid | Ifaty | Ifaty Beach | -23.141979 | 43.617667 | 2007 | Jan-Feb | Wet | Low | NEG | | 0 | 1 | 1 | swab (d) | PMU | qPCR | Weldon & Vences Team | SF State U. | Vredenburg et al. 2012 |
| Subarid | Isalo | Multiple sites, mostly Namazaha Valley | -22.53667 | 45.37483 | 2007 | Jan-Feb | Wet | Mid | NEG | | 0 | 39 | 10 | swab (d) | PMU | qPCR | Weldon & Vences Team | SF State U. | Vredenburg et al. 2012 |
| Subarid | Toliara | Surroundings of Toliara town ♦ | -23.3547 | 43.744833 | 2007 | Jan-Feb | Wet | Low | NEG | | 0 | 32 | 3 | swab (d) | PMU | qPCR | Weldon & Vences Team | SF State U. | Vredenburg et al. 2012 |
| Subarid | Menabe* | Bemanonga, Allée des baobabs ♦ | -20.22651 | 44.43103 | 2012 | Jan | Wet | Mid | NEG | | 0 | 48 | 2 | swab (e) | SE | PCR & qPCR | NMP-Chytrid Emergency Cell | UMB/Imp. C | |
| Subarid | Menabe | Bekonazy | -20.25 | 44.4 | 2013 | Feb | Wet | Mid | NEG | | 0 | 54 | 4 | swab (e) | PMU | qPCR | NMP-Chytrid Emergency Cell | ZSL | |
| Subhumid | Antananarivo | Ricefields and gardens of Institute Pasteur ♦ | -18.90259 | 47.5455 | 2006 | Feb | Wet | High | NEG | | 0 | 20 | UNK | histology | NA | NA | Weldon & Vences Team Team | NWU | Weldon et al. 2008 |
| Subhumid | Antananarivo | Ricefields and gardens of Institute Pasteur ♦ | -18.90259 | 47.5455 | 2006 | Feb | Wet | High | NEG | | 0 | 4 | 2 | swab (d) | PMU | qPCR | Weldon & Vences Team | SF State U. | Vredenburg et al. 2012 |
| Subhumid | Ambohitantely | forest parcel near park administration buildings | -18.19814 | 47.2817 | 2005 | Jan | Wet | High | NEG | | 0 | 29 | UNK | histology | NA | NA | Weldon & Vences Team | NWU | Weldon et al. 2008 |
| Subhumid | Ambohitantely | forest parcel near park administration buildings | -18.19814 | 47.2817 | 2014 | Jan | Wet | High | NEG | | 0 | 30 | 12 | swab (d) | MBPS | qPCR | Bletz Team | JMU | |
| Subhumid | Antoetra | Ankaramasina | -20.806 | 47.3158 | 2014 | Jan | Wet | High | NEG | | 0 | 19 | 5 | swab (d) | MBPS | qPCR | Bletz Team | JMU | |
| Subhumid | Antoetra | Farimazava | -20.82285 | 47.33219 | 2014 | Jan | Wet | High | NEG | | 0 | 8 | 4 | swab (d) | MBPS | qPCR | Bletz Team | JMU | |
| Subhumid | Antoetra | Fohisokina | -20.70517 | 47.28733 | 2011 | Oct | Wet | High | NEG | | 0 | 50 | 2 | swab (e) | SE | PCR | NMP-Chytrid Emergency Cell | UMB | |
| Subhumid | Antoetra | Fohisokina | -20.70517 | 47.28733 | 2012 | Mar | Dry | High | NEG | | 0 | 50 | 2 | swab (e) | SE | PCR | NMP-Chytrid Emergency Cell | UMB | |
| Subhumid | Antoetra* | Fohisokina* | -20.70517 | 47.28733 | 2012 | Oct | Wet | High | POS | 21 | NA | 50 | 1 | swab (e) | SE | PCR & qPCR | NMP-Chytrid Emergency Cell | UMB/Imp. C | |
| Subhumid | Antoetra | Soamazaka | -20.74795 | 47.2952 | 2014 | Jan | Wet | High | POS | 2.88 | 11.11 | 9 | 1 | swab (d) | MBPS | qPCR | Bletz Team | JMU | |
| Subhumid | Itremo | Ambatofinandrana Massif[a] | -20.59333 | 46.56333 | 2008 | Nov | Wet | High | NEG | | 0 | 56 | 12 | swab (d) | 5PAP | PCR | Andreone Team | UMB | Crottini et al. 2011 |
| Subhumid | Montagne d' Ambre | Ankorika, Namakia, & Grand Lac ♦ | -12.556 | 49.165 | 2008 | Feb | Wet | High | NEG | | 0 | 180 | UNK | swab (e) | PMU | qPCR | Vences Team | ZSL | |

*Supplementary Data 2*

*Amphibian chytrid fungus infectivity*

Exposure trials provide preliminary data on the infectivity of selected species to *Bd*. Prior to exposure all frogs tested negative for *Bd,* and all control animals remained negative throughout the duration of the experiment. At least one individual of all species, with the exception of *Guibemantis liber,* became infected with *Bd*. For *Mantidactylus betsileanus* and *Ptychadena mascareniensis* 11 of the 12 individuals exposed to *Bd* became infected, and the mean infection intensity on day 20 was 106.8 ZE and 1.7 ZE respectively. For *Boophis madagascariensis*, by day 20, only one of nine exposed to *Bd* became infected. This individual had an infection intensity of 8.9 ZE. For *B. viridis*, only one individual survived after day 6 of the experiment, and this individual remained infected through the experiment, with an infection intensity of 4.4 ZE on day 20 (Table 3). Both exposed individuals of *H. betsileo* became infected with *Bd,* and on day 20 the mean infection intensity was 48.9 ZE. The single individual of *G. liber* exposed to *Bd* did not become infected. Three individuals, two *B. viridis* (prior to day 6) and one *B. madagascariensis* (prior to day 20) died during the trials. Only one of the *B. viridis* mortalities was shown to be infected with *Bd*.

*Supplementary Table 2: Bd* infection prevalence throughout the conducted exposure trials. Infection prevalence for Day 0, 6, 15 and 20 and mean zoospore intensities on day 20 for each frog species are given. Sample size per treatment for each species is also provided.

| **Species** | **# exposed (# control)** | ***Bd* infection prevalence** | | | | **Mean ZE day 20 (± SD)** |
|---|---|---|---|---|---|---|
| | | **Day 0** | **Day 6** | **Day 15** | **Day 20** | |
| *Mantidactylus betsileanus* | 12 (3) | 0/12 | 9/12 | 10/12 | 11/12 | 106.8 (113.2) |
| *Ptychadena mascareniensis* | 12 (3) | 0/12 | 9/12 | 11/12 | 11/12 | 1.7 (2.5) |
| *Boophis madagascariensis* | 9 (1) | 0/9 | 1/9 | 1/9 | 1/8 | 1.0 |
| *Boophis viridis* | 3 | 0/3 | 1/1 | 1/1 | 1/1 | 4.4 |
| *Heterixalus betsileo* | 2 | 0/2 | 1/2 | 2/2 | 2/2 | 48.9 (68.1) |
| *Guibemantis liber* | 1 | 0/1 | 0/1 | 0/1 | 0/1 | - |

*Supplementary Methods 1*

Exposure trials were set up to assess the ability of *Bd* to infect Malagasy frog species. Six different species of Malagasy amphibians (*Boophis madagascariensis*, *B. viridis, Heterixalus betsileo, Guibemantis liber, Mantidactylus betsileanus, Ptychadena mascareniensis*) were collected in Andasibe, central eastern Madagascar, in July 2009 and exported to the North-West University (NWU) in Potchefstroom, South Africa. These trials were conducted under the ethics permit no. NWU-00013-10-S4 issued by the NWU Research Ethics Committee. All specimens were housed in enclosures, separated by species, for a 12-day acclimation period. The enclosures were fitted with Sphagnum moss, a PVC pipe shelter, and a water bath. Each enclosure was moistened twice a day and cleaned every 4 days, and frogs were fed crickets every three days.

After the 12-day acclimation, individuals were assigned to one of the two treatments: “control” or “exposure to *Bd*” (see supplementary table 2 for details of treatment grouping). Because of low individual sample sizes for some species, control groups were not available for all species. In the “exposure” group, frogs were exposed to a 5-day regiment of *Bd*GPL (strain MG04 isolated from *Amietia fuscugula*, Western Cape, South Africa). Each day, frogs were bathed in 100 ml of water containing 1.5 x $10^3$ zoospores/ml for 3 hours. Control animals were treated with a sham bath of water containing an equal dose of tryptone broth (without *Bd*). Determining if Malagasy frogs can be infected with *Bd* can be tested conclusively without control groups, but due to the lack of controls we cannot establish whether or not mortality might have been confounded by husbandry conditions. Each group (exposed or control) was co-housed by species in enclosures throughout a 20-day trial.

All frogs (control and experimental groups) were routinely swabbed on days 0 (day before treatment), six (day after last treatment), 15 and 20. These samples as well as those of animals that died during the experiment were analysed for *Bd* with qPCR (Boyle et al. 2004) at the National Zoological Gardens, Pretoria, South Africa.
